# Sternal Cleft associated with Patent Ductus Arteriosus, Atrial Septal Defect, and Subglottic Hemangioma: A rarity

**Published:** 2014-04-01

**Authors:** Naser Sadeghian, Alireza Mirshemirani, Irandokht Sadeghian

**Affiliations:** Pediatric Surgery Research Center, Shahid Beheshti University of Medical Sciences, Tehran, Iran.

**Keywords:** Sternal cleft, Subglottic hemangioma, PHACES syndrome

## Abstract

We present a 2-day-old female neonate with cleft of the upper sternum, patent ductus arteriosus (PDA), atrial septal defect (ASD), and subglottic hemangioma. Dimensional and Doppler echocardiography, abdominal ultrasonography, and imaging were performed. She underwent a surgical repair of sternal cleft in neonatal life. After 8 months, she developed respiratory distress, apnea due to subglottic hemangioma. She underwent urgent tracheostomy. Subglottic hemangioma was treated with the KTP532 laser.

## INTRODUCTION

Sternal cleft is a rare congenital anomaly which results from failure of fusion of the 2 lateral mesodermal sternal bars during 8th week of gestation.[1] It is often associated with aplasia cutis congenita (ACC) at sternum. Subglottic hemangioma is also a rare entity.[2] Numerous treatment modalities have been advocated like systemic corticosteroids, tracheostomy, carbon dioxide laser, or interferon alfa-2a. The coexistence of subglottic hemangioma and sternal cleft in presence of cardiac anomalies may pose diagnostic and treatment challenges especially when subglottic hemangioma is revealed on subsequent presentation. We herein report a rare association of sternal cleft and ACC with subglottic hemangioma, PDA, and ASD.

## CASE REPORT

A 2-day-old, term female neonate, presented with a U-shaped cleft of the upper half of the sternum, with central area of ulcerated area of overlying skin through which pulsating heart was visible (Fig. 1). There was no history of maternal infection or medication during early pregnancy. Dimensional and Doppler echocardiography showed a sternal cleft, PDA, ASD, and a normal aortic arch. Abdominal ultrasonography was normal. The patient underwent surgery at 3rd day of life. The necrotic fibrous tissue (ACC) on the sternal cleft area was completely resected without opening the pericardium. Fasciocutaneous flaps were raised on either sides and both sternal bars were approximated with 2/0 PDS and covered by fasciocutaneous flaps.

**Figure:**
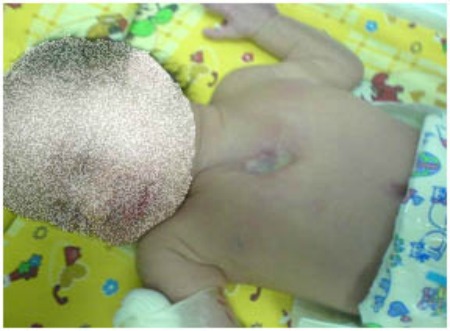
Figure 1:Sternal cleft before operation.

After 8 months, she was readmitted because of the severe respiratory distress and stridor. On examination, there was a hemangioma involving the lower lip, oral mucosa, and right parotid region. Urgent spiral CT-scan of head and neck revealed subglottic hemangioma. A tracheostomy was performed to relieve the respiratory distress. She underwent laryngoscopy and bronchoscopy a week later and subglottic hemangioma was treated by KTP532 laser. Tracheostomy tube was removed subsequently. She had no respiratory distress or subglottic hemangioma at four year follow-up (Fig. 2).

**Figure:**
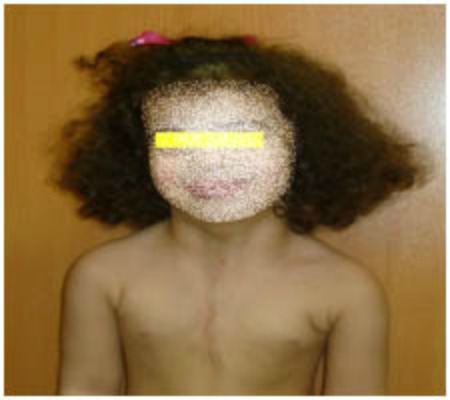
Figure 2:The patient at 4 year follow-up.

## DISCUSSION

A cleft sternum (also known as sternal fissure or bifid sternum) is a rare congenital anomaly with unknown etiology.[1] Majority of the cases of sternal clefts are reported in female patients. Jose et al have reported 8 cases of congenital sternal clefts including six female and two male patients.[2] Sternal clefts can be superior, inferior, and complete. The superior cleft, as seen in the index case, is the frequent one. Superior defects are usually U-shaped as compared to inferior ones which are V-shaped. Sternal cleft is also a part of PHACES (Posterior fossa malformations, Hemangiomas, Arterial malformations, Coarctation of the aorta, Cardiac defects, Eye abnormalities, and Sternal clefts) syndrome. Mazzie et al reported a female neonate with upper sternal cleft and ACC, associated with cardiovascular anomalies, and hemangioma involving face, mouth, and upper trachea causing stenosis of glottis.[3] They considered their patient a forme fruste of PHACES syndrome. Our patient also had upper sternal cleft, with cardiac anomalies, and hemangioma involving the same pattern as the case of Mazzie et al. Thus our case can also be considered as forme fruste of PHACES syndrome.

Treatment of sternal cleft is surgical apposition of sternal bars. The surgery is usually performed in neonatal period when sternal bars and chest wall are most compliant. Treatment is neonatal life is associated with good functional and cosmetic outcome as found in the index case.[2,3] To conclude, any patient with sternal cleft anomaly should carefully be evaluated for cardiac anomalies, and airway hemangioma. PHACES syndrome should be considered in any infant of sternal cleft presenting with a large and segmental facial hemangioma.

## Footnotes

**Source of Support:** Nil

**Conflict of Interest:** None declared

